# A Parametric Logarithmic Image Processing Framework Based on Fuzzy Graylevel Accumulation by the Hamacher T-Conorm

**DOI:** 10.3390/s21144857

**Published:** 2021-07-16

**Authors:** Constantin Vertan, Corneliu Florea, Laura Florea

**Affiliations:** LAPI—The Image Analysis and Processing Laboratory, Politehnica University of Bucharest, 060042 Bucharest, Romania; corneliu.florea@upb.ro (C.F.); laura.florea@upb.ro (L.F.)

**Keywords:** logarithmic image processing, image processing models, fuzzy image processing, T-conorms

## Abstract

It has been proven that Logarithmic Image Processing (LIP) models provide a suitable framework for visualizing and enhancing digital images acquired by various sources. The most visible (although simplified) result of using such a model is that LIP allows the computation of graylevel addition, subtraction and multiplication with scalars within a fixed graylevel range without the use of clipping. It is claimed that a generalized LIP framework (i.e., a parameterized family of LIP models) can be constructed on the basis of the fuzzy modelling of gray level addition as an accumulation process described by the Hamacher conorm. All the existing LIP and LIP-like models are obtained as particular cases of the proposed framework in the range corresponding to real-world digital images.

## 1. Introduction

Logarithmic Image Processing (LIP) models provide a suitable framework for visualizing and enhancing digital images, acquired from various sources and obtained by transmitted/reflected light through absorbing/reflecting media, where the effects are naturally of a multiplicative form. Such an approach was pioneered by the work of Stockham [1], who proposed an enhancement method based on the homomorphic theory introduced by Oppenheim [2,3]. The key to this approach is a homomorphism which transforms the product into a sum (by the use of the logarithm) and thus it allows the use of the classical linear filtering in the presence of multiplicative components.

The underlying initial reason for the introduction of such models has been the necessity to deal with multiplicative phenomena (as in the case of an X-ray image, where the image values represent the transparency/opacity of the real objects). Later, it has been proven that LIP models have a precise mathematical structure and, hence, are suitable for various image processing applications, not necessarily of multiplicative nature. The most used logarithmic representation, evolving from the multiplicative properties of the transmission of light and of the human visual perception, was developed by Jourlin and Pinoli [4,5], under a very well elaborated and rigorous mathematical form. The model has been later extended to color images [6].

The practical importance of the LIP models also comes from another perspective. As one may easily notice, the image functions that are currently used are bounded (taking values in a bounded interval [0,D)). In the course of image processing, the mathematical operations defined on real valued functions, implicitly using the algebra of the real numbers (i.e., on the whole real axis), may produce results that do not belong anymore to the given value interval [0,D)—the only values with physical meaning. Such an approach was discussed for instance in [7] for X-ray image enhancement or in [8] for the creation of high dynamic range images by bracketing. The most recent and comprehensive review of the use of the LIP in various image processing and computer vision applications is presented in [9].

In this paper, we show that a generalized LIP framework (i.e., a parameterized family of LIP models) can be constructed, based on the fuzzy modelling of graylevel addition as an accumulation process described by fuzzy conorms. All the existing LIP and LIP-like models are obtained as particular cases of the proposed framework, under the use of the Hamacher conorm and within the [0,D) range corresponding to real-world digital images. Thus, the paper presents the following claims: (i) a new, fuzzy logic based model for the generation of a parametric set of logarithmic-type image processing models that (ii) generalizes the main existing LIP models and (iii) can provide for some fundamental image processing operations a marginally better performance than the classical LIP.

The remainder of the paper is organized as follows: Section 2 describes the existing LIP models, Section 3 presents the background of the proposed fuzzy logic aggregational approach to graylevel addition and the subsequent construction of the resulting image processing model, which are proven to generalize the existing LIP models, and Section 4 suggests some applications of the proposed parametric LIP model family. The paper ends with some conclusions and suggestions for further research.

## 2. Logarithmic Image Processing Models

In this section, the key points of the existing logarithmic image processing models—the classical model introduced by Jourlin and Pinoli [5,10], and the model introduced by Pătraşcu [11] will be briefly presented, together with the pseudo-LIP model introduced in [12] and two parametric LIP models. This presentation will employ the original notation of the operators, as proposed by their respective authors.

### 2.1. The Classical LIP Model

The classical LIP model, introduced in [5,10], starts by modelling the scalar intensity image by a gray tone function, with values in the bounded domain [0,D). The basic arithmetic operations (addition and subtraction) between two gray tone functions (i.e., images) and the multiplication of the gray tone function via a real-valued scalar are defined in terms of classical operations on R as pixel-level operations. Thus, image addition, subtraction and scalar multiplication are defined by operations on the pixel (scalar) values, denoted generally as *v*.

The addition of gray tones (pixel values) $v_{1}$ and $v_{2}$, denoted by the operator ⨹, is defined as:

(1) $$v_{1}⨹v_{2}=v_{1}+v_{2}-\frac{v_{1}v_{2}}{D}=D\left(\frac{v_{1}}{D}+\frac{v_{2}}{D}-\frac{v_{1}}{D}\frac{v_{2}}{D}\right)$$

The subtraction of (or difference between) gray tones (pixel values) $v_{2}$ and $v_{1}$, denoted by the operator ⨺, is defined as:

(2) $$v_{2}⨺v_{1}=D\frac{v_{2}-v_{1}}{D-v_{1}}=D\frac{\frac{v_{2}}{D}-\frac{v_{1}}{D}}{1-\frac{v_{1}}{D}}.$$

The multiplication of a gray tone (pixel value) *v* by a real scalar λ, denoted by the operator ⨻, is defined as:

(3) $$\lambda ⨻v=D-D{\left(1-\frac{v}{D}\right)}^{\lambda }=D\left(1-{\left(1-\frac{v}{D}\right)}^{\lambda }\right)\;.$$

The key mathematical fundamental of implementing the LIP operations defined above via classical, real-number operations, is the isomorphism between the vector space of gray tone functions $\left((-\infty ,D),⨹,⨻\right)$ and the vector space of real numbers $\left(R,+,\cdot \right)$. The isomorphism is realized through the function T:(−∞,D)→R, defined as:

(4) $$T\left(v\right)=D\log\frac{D}{D-v}=-D\log\left(1-\frac{v}{D}\right).$$

The particular nature of this isomorphism induces the logarithmic character of the mathematical model. In the following, we will consider only the [0,D) part of the functional domain of transform *T* above, since this is the only one in which gray tones correspond to real-world digital images (that is, images with bounded, positive values), similarly to the observation in [13].

Recently, the logarithmic adaptive neighborhood image processing (LANIP) was proposed [14]; this approach is based on the logarithmic image processing (LIP) and on the general adaptive neighborhood image processing (GANIP) [15,16]. The logarithmic part of the model involves the use of the LIP operations instead of the real-axis arithmetic operations. The authors claim an impressive collection of applications arising from the intensity and spatial properties of the human brightness perception that are mathematically modelled and implemented through the combination of logarithmic arithmetics and adaptive selection of the spatial support of the operations.

Very recently, based on the fundamental model described by (4) an asymmetric version was proposed in [17], by translating the isomorphic transformation *T* from Equation (4) along the real axis.

### 2.2. The Homomorphic LIP Model

The logarithmic model introduced by Pătraşcu (presented for instance in [11]) does work with bounded, symmetrical real sets: the gray-tone values of the involved images, defined in [0,D), are linearly transformed into the standard set (−1,1) by:

(5) $$v=\frac{2}{D}\left(u-\frac{D}{2}\right)$$

where u∈[0,D) and v∈(−1,1).

The (−1,1) interval plays the central role in the proposed model: it is endowed with the structure of a linear (moreover, Euclidean) space over the scalar field of real numbers, R. In this space, the addition between two graylevels, $v_{1}$ and $v_{2}$ is defined as:

(6) $$v_{1}⊕v_{2}=\frac{v_{1}+v_{2}}{1+v_{1}v_{2}}$$

The multiplication of a graylevel *v* with a real scalar λ∈R is:

(7) $$\lambda ⊗v=\frac{{\left(1+v\right)}^{\lambda }-{\left(1-v\right)}^{\lambda }}{{\left(1+v\right)}^{\lambda }+{\left(1-v\right)}^{\lambda }}\;.$$

The difference between two graylevels $v_{2}$ and $v_{1}$ is given by:

(8) $$v_{2}⊖v_{1}=\frac{v_{2}-v_{1}}{1-v_{1}v_{2}}.$$

The vector space of graylevels $\left((-1,1),⊕,⊗\right)$ is isomorphic to the space of real numbers $\left(R,+,\cdot \right)$ by the function T:(−1,1)→R, defined as:

(9) $$T\left(v\right)=\log\left(\frac{1+v}{1-v}\right)$$

### 2.3. The Pseudo-LIP Model

Another LIP-like model was introduced in [12] under the name of pseudo-LIP model. The graylevels that are processed are considered within the [0,1) range, which is isomorphic with $R^{+}$ via a transform T:[0,1)→R defined as:

(10) $$T\left(v\right)=\frac{v}{1-v}$$

The transform defined above in (10) is just resembling a logarithmic function (we should note that the functions used for the definitions of the isomorphisms in the classical LIP model (4) and in the homomorphic LIP model (9) are logarithmic functions), thus justifying the pseudo-LIP name.

Under the proposed isomorphism, the addition of graylevels $v_{1}$ and $v_{2}$, denoted by the operator ⊕ is defined as:

(11) $$v_{1}⊕v_{2}=\frac{v_{1}+v_{2}-2v_{1}v_{2}}{1-v_{1}v_{2}},$$

The subtraction of (or difference between) graylevels $v_{2}$ and $v_{1}$ (with $v_{2}\geq v_{1}$), denoted by the operator ⊖ is defined as:

(12) $$v_{2}⊖v_{1}=\frac{v_{2}-v_{1}}{1+v_{1}v_{2}-2v_{1}}.$$

The multiplication of a graylevel *v* by a real scalar λ, denoted by the operator ⊗ is defined as:

(13) $$\lambda ⊗v=\frac{\lambda v}{1+(\lambda -1)v}\;.$$

### 2.4. The Multiparametric LIP

A first parametric LIP (PLIP) model was introduced in [18], by means of the functional modification of the upper range of the values involved in the computations. As such, instead of using the normalization to the upper range values, say *D*, the model proposed in [18] uses a set of functions (γ and *k*) that depend on the upper range of graylevels, *D*, as described in equations below.

(14) $$v_{1}\tilde{⊕}v_{2}=v_{1}+v_{2}-\frac{v_{1}v_{2}}{\gamma (D)}$$

(15) $$\lambda \tilde{⊗}v=\gamma \left(D\right)-\gamma \left(D\right){\left(1-\frac{v}{\gamma (D)}\right)}^{\lambda }\;.$$

The difference between two graylevels $v_{2}$ and $v_{1}$ is given by:

(16) $$v_{2}\tilde{⊖}v_{1}=k\left(D\right)\frac{v_{2}-v_{1}}{k\left(D\right)-v_{1}}.$$

The authors recommend that the γ and *k* functions used in (14) and (16) should be affine functions whose values should be experimentally determined following the optimization of the operations with respect to the given problem.

In order to set up a link between classical LIP and linear arithmetic operation, an extended parametric model, the PLIP model has been further proposed by Panetta et al. [19], by using five parameters, μ, γ, λ, *k* and β, which allow for fine tuning of the classical LIP model, giving users greater control over the result. This model can switch between linear arithmetic operation and classical LIP with various parameters. The basic PLIP operations are defined as follows:

(17) $$v_{1}\tilde{⊕}v_{2}=v_{1}+v_{2}-\frac{v_{1}v_{2}}{\gamma }$$

and, respectively:

(18) $$c\tilde{⊗}v=\lambda -\lambda {\left(1-\frac{v}{\lambda }\right)}^{c}\;.$$

The difference between two graylevels $v_{2}$ and $v_{1}$ is given by:

(19) $$v_{2}\tilde{⊖}v_{1}=k\frac{v_{2}-v_{1}}{k-v_{1}}.$$

It can be shown that the graylevel interval is isomorphic with $R^{+}$ via the transform *T* defined as:

(20) $$T\left(v\right)=\lambda {\left(ln\left(1-\frac{v}{\lambda }\right)\right)}^{\beta }$$

One may notice that the proposed multi-parametrization is cumbersome, not related to any physical or theoretical setup and is intrinsically subject to unmentioned constraints.

### 2.5. The Gigavision-Camera LIP Model

More recently, Deng [20] proposed a generalization of the classical LIP model of Jourlin and Pinoli as a result of the interpretation of the newly proposed Gigavision sensor model [21]. At first glance, the paper suggests a bridge between two, apparently different image models and proves that the classic LIP can be obtained under particular conditions from the functions that describe the Gigavision sensor.

In the Gigavision camera, each pixel of the sensor consists of *N* identical subpixels, each of them receiving the same amount of light, say λ/N. The output of the sensor, at each pixel, is the number of subpixels that have received at least a fixed amount *T* of incoming light, which is

(21) $$v=\Phi_{T}^{-1}\left(\lambda \right)=N\left(1-\frac{\Gamma (T,\lambda /N)}{\Gamma (T)}\right)$$

In the equation above, Γ(x) and Γ(x,y) are the complete and incomplete Gamma functions. The bridge between the models comes from the consideration of the number of graylevels (maximal graylevel range) from the LIP equal to the number of the subpixels of the Gigavision sensor model (that is, D=N). The proposed generalization simply accommodates the use of different number of subpixels for various pixels being processed, under various Gigavision subpixel threshold values. Particularly, for T=1, one obtains the classical LIP isomorphism; for T>1, the transforms defining the model are not analytical.

### 2.6. The Spherical Color Coordinates Model

Within the framework of a very specific application (image compositing), in [22], Grundland et al. propose new operations for colors, such that the color space becomes an ordered field. The original RGB color components and mapped into R by a new isomorphism, that has a logarithmic nature and has a fuzzy logic background, being the generator of the Frank T-conorm. The proposed isomorphism is:

(22) $$T\left(v\right)=1-\log_{\lambda }\left(1+\frac{\lambda -1}{\lambda^{v\lambda /(\lambda -1)}}\right).$$

The original color components are scaled into the (−1,1) interval; all the basic mathematical operations (addition, subtraction, scalar multiplication) are defined by the proposed isomorphism and applied for image blending, in conjunction with contrast enhancement, saliency computation and multiscale processing. This application is further discussed in Section 4.4.

## 3. Fuzzy Aggregation of Graylevels

All image processing algorithms must deal with the imprecision and vagueness that naturally arise in the digital representation of visual information. Noise, quantization, sampling errors, and the tolerance of the human visual system are some of the causes of this imprecision. This strongly suggests that fuzzy models could be used for dealing with the mentioned challenges, as proven by the important number of reported fuzzy image processing applications. Image content fuzziness was thoroughly investigated over the last two decades [23,24,25] and fuzzy image processing applications now range from image enhancement to segmentation and recognition.

Another quality of fuzzy logic, less exploited in the literature, is its ability to model accumulation of items (such as the image graylevels) within fixed bounds. The addition operation of the graylevels can be viewed as the accumulation of the contribution of each individual pixel into a global contribution (the result of the addition). Thus, the addition between the graylevels is modelled as a “stockpile” and described by a sum operation. This is the classical, real-number algebra operation, that clearly may exceed the interval of values with physical meaning. We propose here to view the above accumulation of pixel values as a fuzzy interaction model that groups together the individual pixel contributions (an idea that was previously used in the construction of fuzzy histograms in the context of content-based image retrieval [26,27]). Such an accumulation process (or aggregation of individual entities) is modelled at the most simple level in the fuzzy theory by a fuzzy T-conorm [28].

### 3.1. Fuzzy T-Conorms

Formally, any fuzzy T-conorm, denoted by *S*, applied on the fuzzy values *a* and *b*, is defined as the dual of its associated T-norm *T* [28], such that:

(23) S(a,b)=1−T(1−a,1−b),∀a,b∈[0,1].

In a more theoretical manner, any T-norm can also be constructed by an additive generator function f:[0,1]→R, a function that is decreasing and has the property that f(1)=0. The T-norm is defined ∀a,b∈[0,1] as:

(24) $$T\left(a,b\right)=f^{-1}\left(f(a)+f(b)\right).$$

It follows that the associated T-conorm *S* is given, ∀a,b∈[0,1], by:

(25) $$S\left(a,b\right)=1-f^{-1}\left(f(1-a)+f(1-b)\right).$$

Over the time, several T-conorms have been proposed, such as the ones introduced by Zadeh, Lukasiewicz, Hamacher, etc. [28]. In the continuation of this work, the use of the Hamacher form, introduced in the late 1970s, will be the solely retained. The Hamacher T-conorm [28] is obtained from the following parametric additive generator function $f=f_{H,p}$, with $p\in R^{+}$:

(26) $$f_{H,p}\left(x\right)=\left\{\begin{array}{c} \frac{1-x}{x},\;p=0\\ \log\frac{p+(1-p)x}{x},\;p>0 \end{array}\right.$$

Obviously, one can easily compute the inverse of the Hamacher parametric additive generator function from Equation (26) above, obtaining:

(27) $$f_{H,p}^{-1}\left(x\right)=\left\{\begin{array}{c} \frac{1}{x+1},\;p=0\\ \frac{p}{e^{x}-\left(1-p\right)},\;p>0 \end{array}\right.$$

### 3.2. Hamacher T-Conorm Induced Parametric LIP

It can be considered that the original graylevels *g* from an image can be transformed into fuzzy values by a typicality approach, normalizing all the values by the upper end of the graylevel range, *D*, namely v=g/D. The fuzzy number *v* obtained following this approach measures how close (or typical, or representative) is the given graylevel *g* with respect to white (the brightest graylevel). This type of fuzzification of the graylevel is the most similar with respect to the notion of “gray tone function”, introduced in the classical LIP model. Any other fuzzification procedure may be applied on *g*, but discussing this matter is beyond the scope of the current contribution.

As a result of the above fuzzification of the graylevels, the addition of any two graylevels can be defined as the fuzzy Hamacher T-conorm of their fuzzified values, say $v_{1}$ and $v_{2}$. The expression holds for all $v_{1},v_{2}\in \left[0,1\right)$:

(28) $$v_{1}⨹{\;}_{p}v_{2}=S\left(v_{1},v_{2}\right)=1-f_{H,p}^{-1}\left(f_{H,p}\left(1-v_{1}\right)+f_{H,p}\left(1-v_{2}\right)\right)$$

The operation in (28) is called the generalized addition. Similarly to the way of obtaining the definition (3) in the classical LIP model, as explained in [10] (following the generalization of an inductive approach), the scalar multiplication of a fuzzified graylevel $v\in \left[0,1\right),\;\forall \alpha \in R^{+}$ can be defined as:

(29) $$\alpha ⨻{\;}_{p}vs.=S\left(\underset{\alpha \;times}{\underset{︸}{v,\ldots ,v}}\right)=1-f_{H,p}^{-1}\left(\sum_{\alpha \;times}f_{H,p}\left(1-v\right)\right)=1-f_{H,p}^{-1}\left(\alpha f_{H,p}\left(1-v\right)\right).$$

The replacement of the analytical expression of the Hamacher generator defined in (26) in the equations above produces the analytical form of the generalized parametric graylevel addition and scalar multiplication, $\forall v_{1},v_{2}\in \left[0,1\right)$, $\forall \alpha ,p\in R^{+}$:

(30) $$\alpha ⨹{\;}_{p}vs.=\left\{\begin{array}{c} 1-\frac{\left(1-v_{1}\right)\left(1-v_{2}\right)}{1-v_{1}v_{2}},\;p=0\\ 1-\frac{\left(1-v_{1}\right)\left(1-v_{2}\right)}{1-\left(1-p\right)v_{1}v_{2}},\;p>0 \end{array}\right.$$

(31) $$\alpha ⨻{\;}_{p}vs.=\left\{\begin{array}{c} \frac{\alpha v}{1-v+\alpha v},\;p=0\\ \frac{1-{\left(\frac{1-(1-p)v}{1-v}\right)}^{\alpha }}{1-p-{\left(\frac{1-(1-p)v}{1-v}\right)}^{\alpha }},\;p>0 \end{array}\right.$$

The detailed demonstrations for obtaining Equations (30) and (31) are presented in Appendixes Appendix A and Appendix B, respectively. One can easily check that the basic properties for addition ($v_{1}⨹{\;}_{p}v_{2}=v_{2}⨹{\;}_{p}v_{1}$, $v⨹{\;}_{p}0=v$, $v⨹{\;}_{p}1=1$) and multiplication ($1⨻{\;}_{p}vs.=v$, $0⨻{\;}_{p}vs.=0$) hold for the new operations defined in (30) and (31).

The graylevel subtraction $v_{1}⨺{\;}_{p}v_{2}$ can be introduced in a similar manner, under the constraint that $v_{1}\geq v_{2}$, by:

(32) $$v_{1}⨺{\;}_{p}v_{2}=\frac{v_{1}-v_{2}}{1+\left(1-p\right)v_{1}v_{2}+\left(p-2\right)v_{2}}$$

If one relates the subtraction operation to the classical LIP model, where the subtraction operation can issue both positive and negative results, it is obvious that the proposed FLIP subtraction does not. This is related to a more complex question: what is the nature of the graylevel difference? Is this difference a graylevel, or does it have another, different, nature (such as the edge intensity)? Through this paper, the graylevel difference is seen as another graylevel, and thus, is constrained to positive values (that is, we compute $v_{1}⨺{\;}_{p}v_{2}$ only if $v_{1}\geq v_{2}$). If needed for applications that require negative values (such as Canny edge extraction), one can redefine the subtraction for any pair of values as follows:

(33) $$v_{1}⊖v_{2}=\left\{\begin{array}{c} v_{1}⨺{\;}_{p}v_{2},\;if\;v_{1}\geq v_{2},\\ -\left(v_{2}⨺{\;}_{p}v_{1}\right),\;if\;v_{2}>v_{1}, \end{array}\right.$$

The basic operations defined in Equations (30)–(32) are linked to the real axis by the generator function (fundamental isomorphism) of the corresponding class of LIP models, given by:

(34) $$T\left(v\right)=f_{H,p}\left(1-v\right)=\left\{\begin{array}{c} \frac{v}{1-v},\;p=0\\ \log\frac{p+(1-p)(1-v)}{1-v},\;p>0 \end{array}\right.\;\mathrm{with}\;p\in R^{+}.$$

This set of models, parametrized by *p*, is named Fuzzy Logarithmic Image Processing model set and denoted by FLIP.

**Figure 1:**
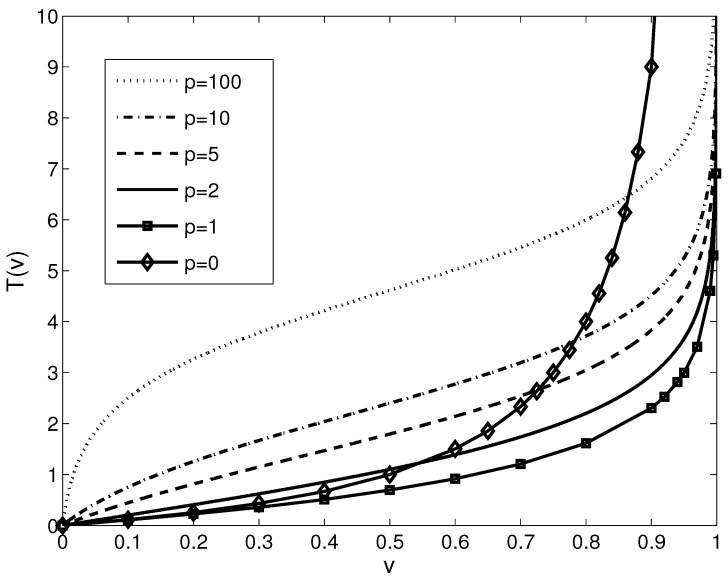
Plot of the fundamental isomorphisms for some of the discussed LIP models particularized from the proposed FLIP: pseudo-logarithmic model (continuous line with diamond marks, p=0), Jourlin and Pinoli’s classical model (4) (lower continuous line with square marks, p=1), Pătraşcu’s model (9) (continuous line, p=2)), and new LIP models (dashed line, p=5, dash-dotted line, p=10 and upper dotted line, p=100).

The generator function $f_{H,p}$ is a bijective function and since it is strictly monotonic, it thus becomes more than an isomorphism; it becomes a homeomorphism. Still, in order to keep the same expression as in the various references used for comparison, the function in (34) will be denoted as an isomorphism. Indeed, the isomorphism in (34) is parametrized by the parameter *p*; specific choices for *p* are presented in Table 1 and graphically shown in Figure 1.

The proof for obtaining the classical LIP, the homomorphic LIP and the pseudo-logarithmic model as particular cases of the proposed FLIP, with *p* parameters chosen according to the values in Table 1 is straightforward. Still, it must be taken into account the fact that for the proposed FLIP, the graylevel range is [0,1], and thus the maximal allowed graylevel becomes D=1, value which must be taken into account when evaluating the equivalence of (30) and (31) with the classical LIP addition (1) and multiplication (3).

One might notice that the result of the addition of any two graylevels increases with respect to the order of the FLIP model, and the difference between the graylevels decreases with respect to the order of the FLIP model.

## 4. Applications

Although the main contribution of this paper is theoretical, as it presents an unifying framework to approach most LIP models under the proposed FLIP, on the practical side several application may be developed. A few obvious applications, such as dynamic range enhancement, noise reduction and edge detection based on linear derivative filtering will be briefly discussed in the following subsections.

### 4.1. Dynamic Range Enhancement

The problem of dynamic range enhancement was addressed (within the classical LIP framework, i.e., FLIP with p=1) in [5], proposing an optimal derivation (analytical proof) of a single scalar multiplication constant of the entire image that maximizes the overall dynamic range. The problem can be expressed as follows: for a given image *f*, find the real scalar α, such that the dynamic range obtained by graylevel amplification (multiplication by the α scalar) is maximized. As proposed in the framework of the classical LIP in [4], the analytical solution for α was:

(35) $$\alpha_{0}=\frac{\ln\left(\frac{\ln(1-\max f)}{\ln(1-\min f)}\right)}{\ln(1-\min f)/(1-\max f)}.$$

For the implementation of the multiplication operation by the scalar α will be used the generalized expression of FLIP scalar multiplication from (31); the problem can be now formulated as to maximize, for the given image *f*, the dynamic range defined as:

(36) $$DR\left(\alpha ,p,f\right)=\alpha ⨻{\;}_{p}\max f-\alpha ⨻{\;}_{p}\min f.$$

The FLIP form of the dynamic range is obtained by replacing in Equation (36) the $⨻{\;}_{p}$ operation with its analytical form from (31); one obtains (after minor algebraic simplifications, which are explained in Appendix C) that:

(37) $$DR\left(\alpha ,p,f\right)=\frac{p}{1-p-\pi (\max f)}-\frac{p}{1-p-\pi (\min f)}.$$

where

(38) $$\pi \left(x\right)={\left(\frac{1-(1-p)x}{1-x}\right)}^{\alpha }.$$

Determining the maximum achievable dynamic range for a given image *f* means to optimize DR(α,p,f) with respect to both α and *p*. Obviously, there is no analytic solution for the required optimization, such that numerical optimization was used instead. Simple tests show that one might expect, at least in some conditions, that the FLIP with p>1 performs better than the classical LIP. We shall denote in the following by $p_{0}$ the order of the best FLIP model achieving maximal dynamic range for a given image *f*, that is:

(39) $$p_{0}=\arg\max_{p}DR\left(\alpha ,p,f\right).$$

**Figure 2:**
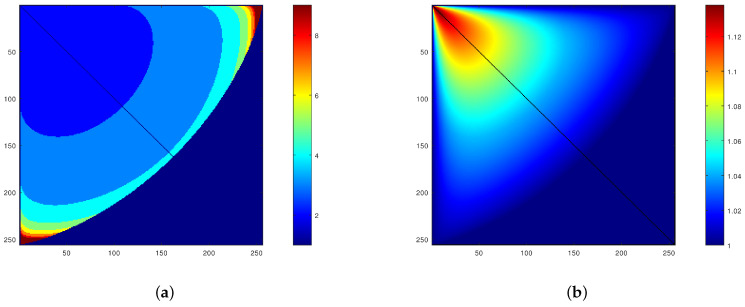
(**a**) Order $p_{0}$ of the FLIP model that achieves optimal dynamic range as a function of the minimal (horizontal axis) and maximal (vertical axis) values within the image (data is obviously symmetric with respect to the diagonal). (**b**) Ratio $DR\left(\alpha ,p_{0},f\right)/DR\left(\alpha_{0},1,f\right)$ of the optimal dynamic range of the FLIP model to the optimal dynamic range of the classical LIP model as a function of the minimal (horizontal axis) and maximal (vertical axis) values within the image (data is obviously symmetric with respect to the diagonal). The parameter and ratio values are color-coded.

Testing of the optimal solution for (37) was performed extensively, over the entire range of possible image graylevels. Figure 2a,b show the most important results: $p_{0}$, the order of the FLIP model that is the best (Figure 2a) and the ratio $DR\left(\alpha ,p_{0},f\right)/DR\left(\alpha_{0},1,f\right)$ (Figure 2b). The ratio of the best FLIP dynamic range to the best classical LIP dynamic range is mostly bigger than 1, showing that in 67% of the image cases, one can achieve a bigger dynamic range using a FLIP with p>1. The dynamic range increase ranges from 0.1% to 100%, with an average of 7.5%.

**Figure 3:**
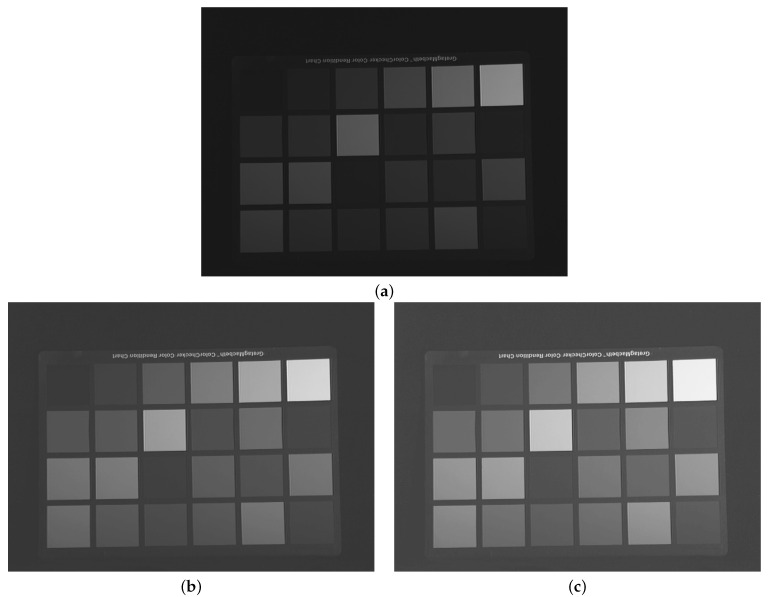
From top to bottom: (**a**) original underexposed image; (**b**) enhanced image (DR=0.7) under the classical LIP model (FLIP with p=1); (**c**) enhanced image (DR=0.71) under the proposed FLIP model (p=5). The original image in (**a**) is taken from the Data for Computer Vision and Computational Colour Science set, described in [29].

The images from Figure 3 show the result of such a dynamic range enhancement applied on the luminance component of a typical poorly illuminated image, taken from the Data for Computer Vision and Computational Colour Science set, described in [29].

### 4.2. Average-Based Noise Reduction

The simplest linear noise reduction method is the averaging operation, suited for white, additive, Gaussian noise (WAGN). It is known that the linear averaging provides the highest noise reduction from any linear filter with fixed weights. Replacing the linear averaging with FLIP additions and multiplications can provide an extra performance with respect to the classical LIP, using models with p>2. A simple experiment was performed by applying the arithmetic averaging, implemented with the FLIP models within a 3×3 filtering window, sliding across various natural images corrupted by WAGN. The FLIP averaging operation at pixel (i,j) within the chosen 3×3 filtering window is described in Equation (40) below:

(40) $$\begin{array}{cc} Average_{p}\left(i,j\right) & =\left(\frac{1}{9}⨻{\;}_{p}f\left(i-1,j-1\right)\right)⨹{\;}_{p}\left(\frac{1}{9}⨻{\;}_{p}f\left(i-1,j\right)\right)⨹{\;}_{p}\left(\frac{1}{9}⨻{\;}_{p}f\left(i-1,j+1\right)\right)⨹{\;}_{p}\\ \; & \left(\frac{1}{9}⨻{\;}_{p}f\left(i,j-1\right)\right)⨹{\;}_{p}\left(\frac{1}{9}⨻{\;}_{p}f\left(i,j\right)\right)⨹{\;}_{p}\left(\frac{1}{9}⨻{\;}_{p}f\left(i,j+1\right)\right)⨹{\;}_{p}\\ \; & \left(\frac{1}{9}⨻{\;}_{p}f\left(i+1,j-1\right)\right)⨹{\;}_{p}\left(\frac{1}{9}⨻{\;}_{p}f\left(i+1,j\right)\right)⨹{\;}_{p}\left(\frac{1}{9}⨻{\;}_{p}f\left(i+1,j+1\right)\right). \end{array}$$

**Figure 4:**
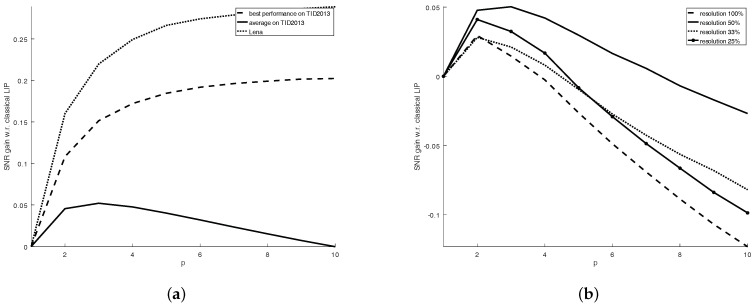
(**a**) Plot of the SNR improvement ($SNR_{p}-SNR_{p=1}$) of the FLIP models with p>1 with respect to the SNR obtained by the classical LIP model (FLIP with p=1) for various images affected by WAGN with a standard deviation of 7. The SNR improvement is defined as the SNR obtained for p>1 minus the SNR obtained for p=1. (**b**) Plot of the SNR improvement ($SNR_{p}-SNR_{p=1}$) of the FLIP models with p>1 with respect to the SNR obtained by the classical LIP model (FLIP with p=1) for various resolutions of images affected by WAGN with a standard deviation of 7.

In the significant majority of cases (as Figure 4a shows for the selection of images from the classical TID2013 image quality database [30]), the filtering performance (in terms of SNR) was increased by using FLIP models with a high *p* value. Figure 4a depicts the difference between the SNR obtained for the image filtered with FLIP-based averaging with p>1 and the SNR obtained for the image filtered with FLIP-based averaging with p=1 (that is, $SNR_{p}-SNR_{p=1}$). The improvements are marginal, but non-negligible. Another quality estimation of the filtering result can be performed by using a non-reference image quality measure. One such measure is BRISQUE (Blind/Referenceless Image Spatial QUality Evaluator) [31], which computes a number within [0,100] claimed to integrate the naturalness losses of images due to various distortion types. As such, the smaller the BRISQUE measure, the more naturally looking (and distortion-free) the image looks. The BRISQUE measure computed for the averaged TID2013 database images shows that an increased value of the parameter *p* can bring a very slight improvement in the naturalness of the image, as the BRISQUE values for the images average filtered by FLIP with p>1 (BRISQUE = 32.45 on average for the entire database for p=10) are consistently smaller than the BRISQUE values for the images average filtered by the classical LIP (BRISQUE = 32.71 on average for the entire database).

The same natural images from TID2013, degraded with WAGN, are filtered by averaging at various resolutions. The results, as presented in Figure 4b, show that the performance increase for the FLIP implementation with p>1 appear mainly when there is a balance between the size of the uniform areas and details within the images. Furthermore, this test shows that the most promising FLIP models are obtained for p≤10. This experiment suggests that the averaging via FLIP can increase the noise reduction performance of the classical LIP.

**Figure 5:**
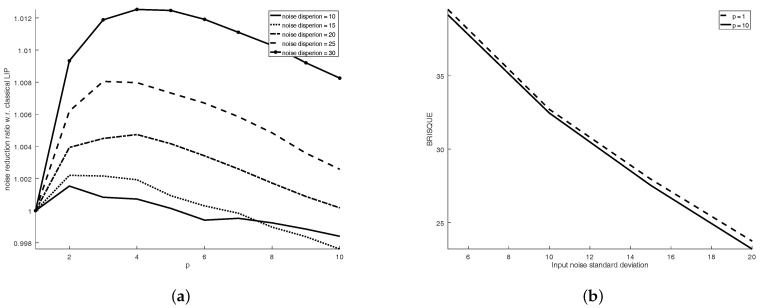
(**a**) Plot of the noise reduction ratio of the FLIP models with p>1 with respect to the classical LIP model (FLIP with p=1) for various WAGN dispersions. (**b**) Average BRISQUE value (smaller is better) of the average filtered images from the TID2013 database for various WAGN dispersions, for images processed by the classical LIP model (p=1) and FLIP with p=10.

For the investigation of the relative noise reduction power of the FLIP models with respect to the classical LIP (i.e., FLIP with p=1) a simple experiment was established. A fully uniform image was degraded by WAGN (with various dispersions) and the image was filtered by the averaging filter implemented according to (40). In the filtered image, the dispersion of the remaining noise was measured and the ratio between the noise dispersion after filtering according to FLIP with p>1 and the noise dispersion after filtering with the classical LIP (p=1) was measured. This noise reduction factor, in dependence to the noise level and the order of the FLIP model is presented in Figure 5, showing that for a significant range of models (i.e., *p* values and noise dispersions, the classical LIP model (i.e., FLIP with p=1) is outperformed by FLIP models with p>1.

### 4.3. Gradient-Based Edge Detection

One of the simplest edge-detection techniques is based on the measuring of the image derivatives, as indication of pixel value variation along specific directions. The usual implementations of such edge detectors are based on linearly implemented first- and second-order derivatives. We will experiment the implementation of such operators under the FLIP model, considering two common examples: the Sobel smoothed gradient as a first-order, linear, classical, derivative edge intensity detector and the Laplacian as a second-order linear, classical, derivative edge intensity detector. Both will be expressed by FLIP operations and their performance will be investigated, following the two important criteria established by Canny: noise rejection and edge localization. It will be shown how the use of the proposed FLIP can increase the performance of a LIP-based derivative edge extraction operator.

The classical Sobel derivative filter is implemented under the FLIP framework as:

(41) $$\begin{array}{cc} Sobel_{H}\left(i,j\right) & =\left(f\left(i-1,j-1\right)⨹{\;}_{p}f\left(i+1,j-1\right)\right)⨹{\;}_{p}\left(f\left(i,j-1\right)⨹{\;}_{p}f\left(i,j-1\right)\right)⨺{\;}_{p}\\ \; & \left(f\left(i-1,j+1\right)⨹{\;}_{p}f\left(i+1,j+1\right)\right)⨹{\;}_{p}\left(f\left(i,j+1\right)⨹{\;}_{p}f\left(i,j+1\right)\right). \end{array}$$

(42) $$\begin{array}{cc} Sobel_{V}\left(i,j\right) & =\left(f\left(i-1,j-1\right)⨹{\;}_{p}f\left(i-1,j+1\right)\right)⨹{\;}_{p}\left(f\left(i-1,j\right)⨹{\;}_{p}f\left(i-1,j\right)\right)⨺{\;}_{p}\\ \; & \left(f\left(i+1,j-1\right)⨹{\;}_{p}f\left(i+1,j+1\right)\right)⨹{\;}_{p}\left(f\left(i+1,j\right)⨹{\;}_{p}f\left(i+1,j\right)\right). \end{array}$$

The LIP-based Laplacian operator was already investigated (for instance in [12]) as a continuation of the work of Deng and Pinoli [32]. The Laplacian used in the experiments is based on the $N_{4}$ neighborhood and is implemented, for the graylevel image *f*, at pixel location (i,j) in the classical, linear case as:

(43) $$\begin{array}{cc} \Delta (i,j) & =\left(f(i,j)-f(i-1,j)\right)+\left(f(i,j)-f(i+1,j)\right)+\\ \; & +\left(f(i,j)-f(i,j-1)\right)+\left(f(i,j)-f(i,j+1)\right). \end{array}$$

and in the FLIP case as:

(44) $$\begin{array}{cc} \Delta (i,j) & =\left(f\left(i,j\right)⨺{\;}_{p}f\left(i-1,j\right)\right)⨹{\;}_{p}\left(f\left(i,j\right)⨺{\;}_{p}f\left(i+1,j\right)\right)⨹{\;}_{p}\\ \; & +\left(f\left(i,j\right)⨺{\;}_{p}f\left(i,j-1\right)\right)⨹{\;}_{p}\left(f\left(i,j\right)⨺{\;}_{p}f\left(i,j+1\right)\right). \end{array}$$

From the derivative values, an edge intensity map is computed as their absolute value and the edge intensity map is adaptively thresholded by a value computed according to the Otsu method.

**Figure 6:**
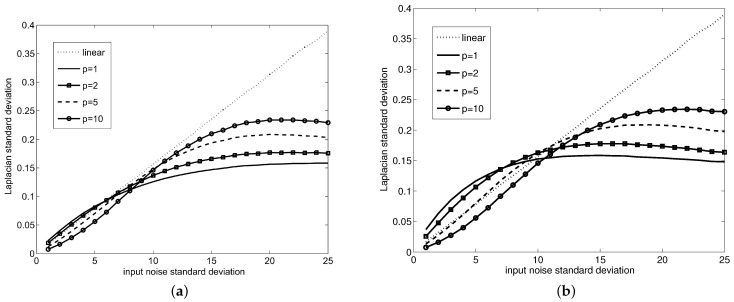
Output WAGN standard deviation vs. input WAGN standard deviation for the classical Laplacian (dotted line), Laplacian under the logarithmic-like model from [12] (p=0) (continuous line with diamond marks), Laplacian under the classical Jourlin-Pinoli logarithmic model [5] (p=1) (continous line), Laplacian under the Pătraşcu logarithmic model [11] (p=2) (dash-dotted line), Laplacian under the proposed FLIP with p=5 (continuous line with square marks) and Laplacian under the proposed FLIP with p=9 (continuous line with circle marks) for two constant images with gray levels (**a**) 75 and (**b**) 150.

The main issue of linear derivative filters is that any noise existing within the image is amplified, in the sense that the variation of the noise is also measured by derivation, sometimes exceeding the intrinsic variations of the original image signal. A linear dependency exists between the noise power within the image and the noise power remaining in the linearly filtered image. We will show with simple experiments that the use of derivative operators based on the newly introduced logarithmic image processing operations can lead to a reduction of the noise power in the filtered image, as compared to the result obtained via classical, real-numbers implementations, or classical LIP implementations of the same derivatives. We will consider the case of a fully uniform image degraded by a white, zero-mean, additive Gaussian noise (WAGN), which is filtered by a Laplacian operator (which is a second-order derivative operator and thus is prone to high noise amplification) implemented under the to paradigms: the classical linear model and the FLIP model. The output noise standard deviation is used as measure of the noise power in both input and filtered images. The plots presented in Figure 6 present the behavior of several FLIP implementations of the Laplacian, for initial uniform images with different values, showing the overall good behavior of the proposed FLIP implementation. The ideal behavior of a noise-stable Laplacian is to have the noise standard deviation curve in Figure 6 as “low” as possible and below the curve corresponding to the classical, linear Laplacian. Namely, the typical behavior is that the Laplacian implemented via the FLIP models exhibits lower output noise dispersion than the classical, linear Laplacian for WAGN with higher standard deviation. In this high noise range, the best performance is achieved by the low-order FLIP models, such as the classical LIP (p=1). In the lower noise range, some of the FLIP models perform worse than the classical, linear Laplacian; still, high-order FLIP models (with p>5) perform better than the classical Laplacian.

The edge localization property is based on the distance between the actual contour and the detected contour, measure in the thresholded edge intensity map. The classical Pratt figure of merit (FOM) is a commonly used tool for the characterization of edge localization. The FOM is computed as:

(45) $$FOM=\frac{1}{\max(D_{E},I_{E})}\sum_{i=1}^{D_{E}}\frac{1}{1+\alpha d_{i}}$$

In the equation above, $D_{E}$ and $I_{E}$ are the detected and the ideal edge pixels and $d_{i}$ is the distance between any detected edge pixel and the closest ideal edge pixel. The FOM is computed for test images composed by vertical/ horizontal edges that separate perfectly uniform regions, affected by white, additive, Gaussian noise with various dispersions.

**Figure 7:**
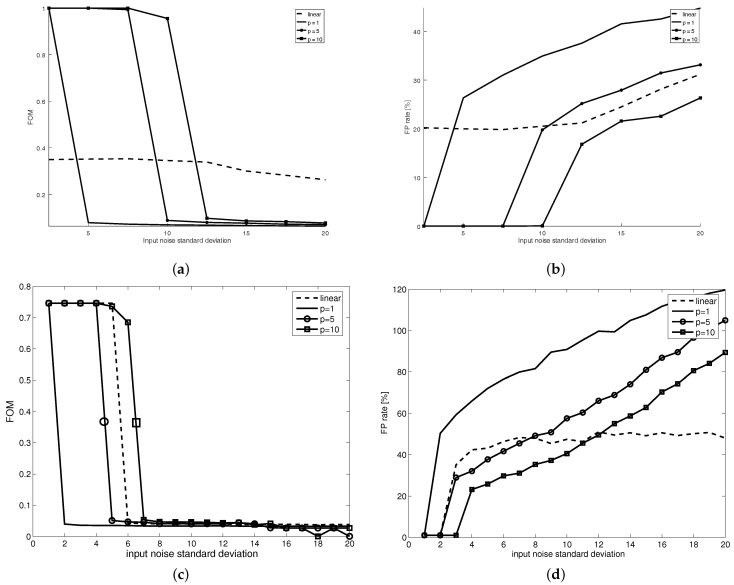
Pratt’s Figure of merit (**a**,**c**) and False Positives rates (**b**,**d**) with respect to the input noise dispersion for the classical, linear model (dashed line), for the classical Jourlin–Pinoli logarithmic model [5] (p=1) (continuous line), for the proposed FLIP with p=5 (continuous line with circular marks) and for the proposed FLIP with p=10 (continuous line with square marks) for a synthetic test image with gray levels difference across the edge of 100 for two standard derivative models: Sobel gradient (**a**,**b**) and Laplacian (**c**,**d**).

As Figure 7 shows, the FOM increases (and the Laplacian performs better) with the increase in the FLIP model order (*p*), for all noise dispersions. Still, the classical, linear Laplacian performance is matched and sometimes exceeded in the case of high-order FLIP models. Figure 7b shows the false positive edge pixel ratio for the same test images; it can be seen that while there is a general, natural increase in the false positive edge pixels ratio induced by the increase in the noise standard deviation, the FLIP models with p>1 can perform better than the classical, linear Laplacian.

**Figure A1:**
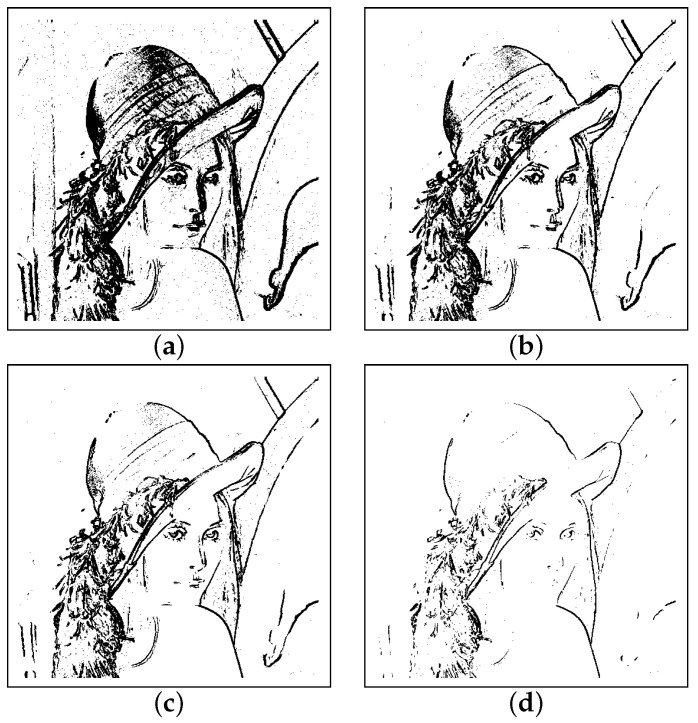
FLIP Laplacian binary edge images of the classical Lena picture computed for various FLIP models: (**a**) p=1 (classical LIP); (**b**) p=5; (**c**) p=10; (**d**) p=50. All the edge maps are obtained by the Otsu thresholding of the Laplacian. One can notice that with the increase in *p* small and un-contrasted contours disappear.

**Figure A2:**
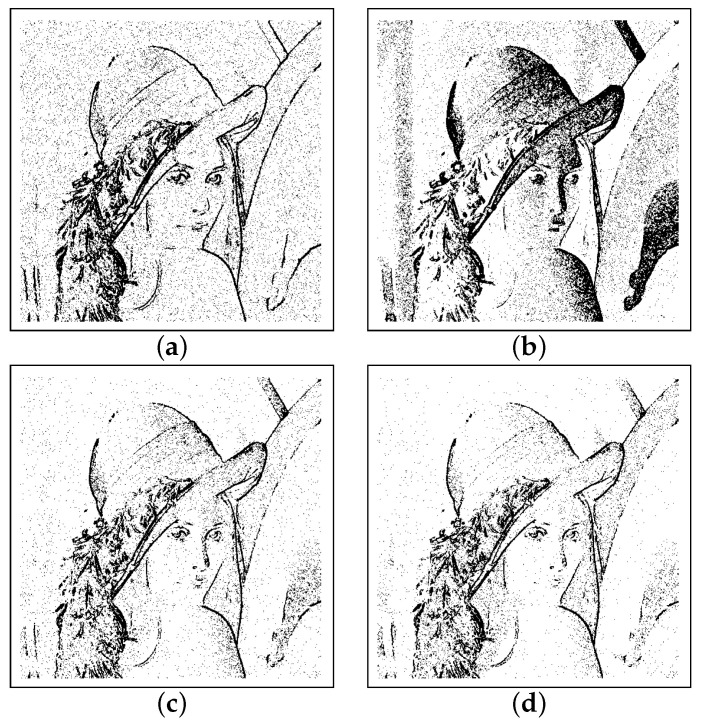
FLIP Laplacian binary edge images of the classical Lena picture, degraded with WAGN of standard deviation 7, computed for various FLIP models: (**a**) classical, linear Laplacian; (**b**) p=1 (classical LIP); (**c**) p=10; (**d**) p=50. All the edge maps are obtained by the Otsu thresholding of the Laplacian. One can notice that with the increase in *p* the false positive edge points produced by the noise decrease (image (**d**)) is much cleaner than image (**b**)).

Finally, some typical visual edge extraction results are shown in Appendix D in Figure A1 and Figure A2; it can be observed that the order of the FLIP model can be seen as a trimming factor for the selection of the most important edges from the picture and as a means for rejecting false positive edges due to noise.

### 4.4. Image Blending

Image blending and High Dynamic Range (HDR) image creation are prime applications in computer graphics; the linear operators, used by their own, have been shown to fade colors and create false colors. There are approaches that, among other pre- and postprocessing steps, replace the linear operators with more suited ones. Such an example is, for instance, the logarithmic HDR, proposed in [8], or the image blending via the spherical color coordinates model, proposed in [22]. These two approaches use logarithmic-type operators for the addition and scalar multiplication of gray or RGB color components.

**Figure 8:**
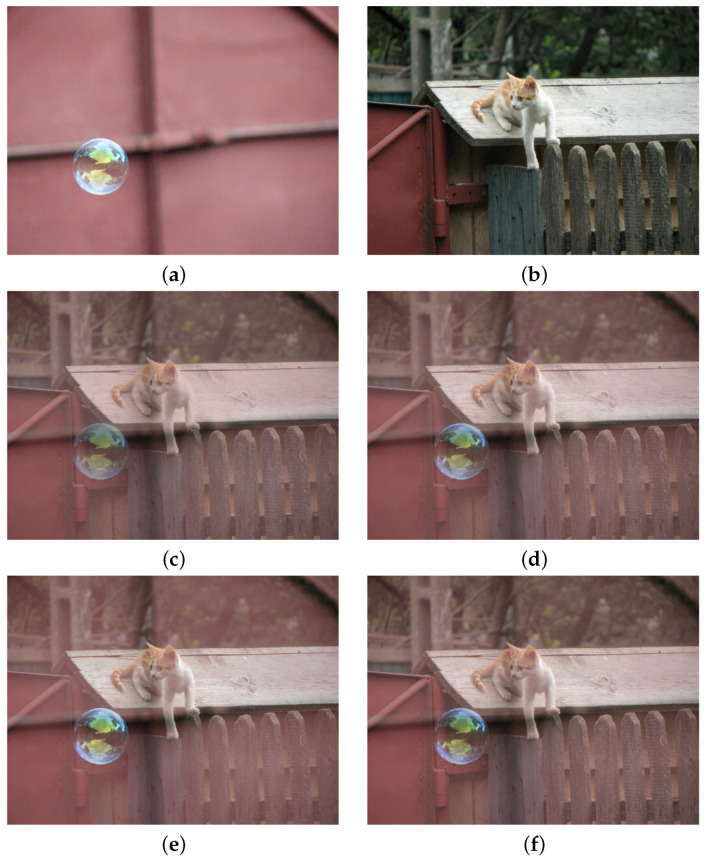
Image blending with equal weights of the original color images in (**a**,**b**). The blending is performed according to: (**c**) linear operations (BRISQUE = 23.60); (**d**) Grundland et al. without contrasting, pyramid processing and saliency (BRISQUE = 26.13); (**e**) FLIP operations with p=1 (classical LIP, BRISQUE = 30.84); (**f**) FLIP operations with p=10 (BRISQUE = 23.72). One can notice that for pixel-based blending, the FLIP models with p=10 offer the best visual results.

In the following example, shown in Figure 8, we compare the simple blending of two images, with equal weights, via linear and various LIP, LIP-like and FLIP models. Namely, the images $I_{1}$ and $I_{2}$ are composed into image *J*, for each color channel independently, as:

(46) $$J=\left(w_{1}⨻I_{1}\right)⨹\left(w_{2}⨻I_{2}\right).$$

Objective evaluation of the blending results can be performed only by the use of a non-reference image quality measure. We will use BRISQUE [31] again for estimating the naturalness of the various blending models. In Figure 8, each blended image was evaluated by BRISQUE (the score is included in the caption) and this evaluation shows that using FLIP operations with p>1 the resulting image looks more natural. Subjective evaluation also shows that the use of the FLIP model with p>1 produce better results than the classical LIP and the results are comparable with the ones proposed in [22] (check for instance the overall appearance of the soap bubble).

## 5. Conclusions

This paper presented a new parametric class of logarithmic models for image processing (LIP), which allows the computation of graylevel addition, subtraction and multiplication with scalars within a fixed graylevel range [0,D) without the use of clipping. This class of models was named FLIP—Fuzzy Logarithmic Image Processing models. We may remind that this work claimed the proposed FLIP as (i) a new, fuzzy logic-based model for the generation of a parametric set of logarithmic-type image processing models that (ii) generalize the main existing LIP models and (iii) can provide for some particular cases a marginally better performance than the classical LIP for various image processing operations.

The derivation of the proposed model is based on the interpretation of graylevel addition as a process of accumulation (or reunion), and thus its modelling by a fuzzy T-conorm (the Hamacher family, in particular). The existing LIP models can be obtained as particular cases of the proposed parametric family. It should be noted that the proposed approach has more limited mathematical properties as compared with the classical LIP models; the most obvious limitation being the fact that the graylevel difference may not yield a negative result. The supplemental choice of a FLIP model (by choosing a particular value of the parameter *p*) may improve the result of some algorithmic approaches (such as the simple presented application of dynamic range enhancement).

## Figures and Tables

**Table 1:** Particular cases of the proposed FLIP framework.

*p*	Model	Fundamental Isomorphism	Domain for *v*
0	pseudo-LIP (10)	$T\left(v\right)=\frac{v}{1-v}$	[0,1)
1	classical LIP (4)	$T\left(v\right)=\log\frac{1}{1-v}$	[0,1)
2	homomorphic LIP (9)	$T\left(v\right)=\log\frac{1+v}{1-v}$	(−1,1)
p>2	new models	$T\left(v\right)=\log\frac{1-(1-p)v}{1-v}$	[0,1)

## Data Availability

Not applicable.

## Appendix A. Derivation of the FLIP Addition

We defined the FLIP addition as the aggregation of the fuzzyfied gray tones $v_{1}$ and $v_{2}$ in Equation (28) as:

(A1) $$v_{1}⨹{\;}_{p}v_{2}=S\left(v_{1},v_{2}\right)=1-f_{H,p}^{-1}\left(f_{H,p}\left(1-v_{1}\right)+f_{H,p}\left(1-v_{2}\right)\right)$$

The equation is particularized by replacing the analytical expressions of the Hamacher generator function $f_{H,p}$ and its inverse $f_{H,p}^{-1}$, as defined in (26) and (27):

(A2) $$f_{H,p}\left(x\right)=\left\{\begin{array}{c} \frac{1-x}{x},\;p=0\\ \log\frac{p+(1-p)x}{x},\;p>0 \end{array}\right.$$

(A3) $$f_{H,p}^{-1}\left(x\right)=\left\{\begin{array}{c} \frac{1}{x+1},\;p=0\\ \frac{p}{e^{x}-\left(1-p\right)},\;p>0 \end{array}\right.$$

We develop the algebraic computation in the case of p=0 and p>0, respectively. First, for p=0:

(A4) $$\begin{array}{cc} v_{1}⨹{\;}_{p}v_{2} & =S\left(v_{1},v_{2}\right)=1-f_{H,p}^{-1}\left(f_{H,p}\left(1-v_{1}\right)+f_{H,p}\left(1-v_{2}\right)\right)\\ \; & =1-f_{H,p}^{-1}\left(\frac{1-(1-v_{1})}{1-v_{1}}+\frac{1-(1-v_{2})}{1-v_{2}}\right)\\ \; & =1-\frac{1}{1+\frac{1-(1-v_{1})}{1-v_{1}}+\frac{1-(1-v_{2})}{1-v_{2}}}=1-\frac{1}{\frac{\left(1-v_{1}\right)\left(1-v_{2}\right)+v_{1}+v_{2}}{\left(1-v_{1}\right)\left(1-v_{2}\right)}}\\ \; & =1-\frac{\left(1-v_{1}\right)\left(1-v_{2}\right)}{1-v_{1}v_{2}}. \end{array}$$

In the case of p>0, we have:

(A5) $$\begin{array}{cc} v_{1}⨹{\;}_{p}v_{2} & =S\left(v_{1},v_{2}\right)=1-f_{H,p}^{-1}\left(f_{H,p}\left(1-v_{1}\right)+f_{H,p}\left(1-v_{2}\right)\right)\\ \; & =1-f_{H,p}^{-1}\left(\log\frac{p+\left(1-p\right)\left(1-v_{1}\right)}{\left(1-v_{1}\right)}+\log\frac{p+\left(1-p\right)\left(1-v_{2}\right)}{\left(1-v_{2}\right)}\right)\\ \; & =1-\frac{p}{e^{\left(\log\frac{p+\left(1-p\right)\left(1-v_{1}\right)}{\left(1-v_{1}\right)}+\log\frac{p+\left(1-p\right)\left(1-v_{2}\right)}{\left(1-v_{2}\right)}\right)}-\left(1-p\right)}\\ \; & =1-\frac{p}{e^{\left(\log\frac{p+\left(1-p\right)\left(1-v_{1}\right)}{\left(1-v_{1}\right)}\right)}\times e^{\left(\log\frac{p+\left(1-p\right)\left(1-v_{2}\right)}{\left(1-v_{2}\right)}\right)}-\left(1-p\right)}\\ \; & =1-\frac{p}{\frac{p+\left(1-p\right)\left(1-v_{1}\right)}{\left(1-v_{1}\right)}+\frac{p+\left(1-p\right)\left(1-v_{2}\right)}{\left(1-v_{2}\right)}-\left(1-p\right)}\\ \; & =1-\frac{p\left(1-v_{1}\right)\left(1-v_{2}\right)}{p\left(1-v_{2}\right)+\left(1-p\right)\left(1-v_{1}\right)\left(1-v_{2}\right)+p\left(1-v_{1}\right)}\\ \; & =1-\frac{\left(1-v_{1}\right)\left(1-v_{2}\right)}{1-\left(1-p\right)v_{1}v_{2}}. \end{array}$$

Finally, we can write compactly that $\forall v_{1},v_{2}\in \left[0,1\right)$, $\forall \alpha ,p\in R^{+}$ (which is Equation (30)):

(A6) $$\alpha ⨹{\;}_{p}vs.=\left\{\begin{array}{c} 1-\frac{\left(1-v_{1}\right)\left(1-v_{2}\right)}{1-v_{1}v_{2}},\;p=0\\ 1-\frac{\left(1-v_{1}\right)\left(1-v_{2}\right)}{1-\left(1-p\right)v_{1}v_{2}},\;p>0 \end{array}\right.$$

## Appendix B. Derivation of the FLIP Scalar Multiplication

We defined the FLIP scalar multiplication as the repeated addition of a fuzzyfied gray tone *v* by a real scalar α in Equation (29) as:

(A7) $$\alpha ⨻{\;}_{p}vs.=S\left(\underset{\alpha \;times}{\underset{︸}{v,\ldots ,v}}\right)=1-f_{H,p}^{-1}\left(\sum_{\alpha \;times}f_{H,p}\left(1-v\right)\right)=1-f_{H,p}^{-1}\left(\alpha f_{H,p}\left(1-v\right)\right).$$

The equation is particularized by replacing the analytical expressions of the Hamacher generator function $f_{H,p}$ and its inverse $f_{H,p}^{-1}$, as defined in (26) and (27). We develop the algebraic computation in the case of p=0 and p>0, respectively. First, for p=0:

(A8) $$\begin{array}{cc} \alpha ⨻{\;}_{p}vs. & =1-f_{H,p}^{-1}\left(\alpha f_{H,p}\left(1-v\right)\right)\\ \; & =1-f_{H,p}^{-1}\left(\alpha \frac{1-(1-v)}{\left(1-v\right)}\right)=1-f_{H,p}^{-1}\left(\alpha \frac{v}{\left(1-v\right)}\right)\\ \; & =1-\frac{1}{\left(\alpha \frac{v}{\left(1-v\right)}\right)+1}=1-\frac{1-v}{\alpha vs.+1-v} \end{array}$$

In the case of p>0, we have:

(A9) $$\begin{array}{cc} \alpha ⨻{\;}_{p}vs. & =1-f_{H,p}^{-1}\left(\alpha f_{H,p}\left(1-v\right)\right)\\ \; & =1-f_{H,p}^{-1}\left(\alpha \log\frac{p+(1-p)(1-v)}{\left(1-v\right)}\right)=1-\frac{p}{e^{\left(\alpha \log\frac{p+(1-p)(1-v)}{\left(1-v\right)}\right)}-\left(1-p\right)}\\ \; & =1-\frac{p}{\alpha \frac{p+(1-p)(1-v)}{\left(1-v\right)}-\left(1-p\right)}=1-\frac{p(1-v)}{\alpha \left(p+(1-p)(1-v)\right)-\left(1-p\right)\left(1-v\right)}\\ \; & =\frac{1-{\left(\frac{1-(1-p)v}{1-v}\right)}^{\alpha }}{1-p-{\left(\frac{1-(1-p)v}{1-v}\right)}^{\alpha }}. \end{array}$$

Finally, we can write compactly that ∀vs.∈[0,1), $\forall \alpha ,p\in R^{+}$ (which is Equation (31):

(A10) $$\alpha ⨻{\;}_{p}vs.=\left\{\begin{array}{c} \frac{\alpha v}{1-v+\alpha v},\;p=0\\ \frac{1-{\left(\frac{1-(1-p)v}{1-v}\right)}^{\alpha }}{1-p-{\left(\frac{1-(1-p)v}{1-v}\right)}^{\alpha }},\;p>0 \end{array}\right.$$

## Appendix C. Derivation of the Formula for the Dynamic Range

The dynamic range of an image *f* is the difference between its maximal and minimal values. After a simple image stretching performed under the FLIP model, the resulting dynamic range is given in Equation (36)

(A11) $$DR\left(\alpha ,p,f\right)=\alpha ⨻{\;}_{p}\max f-\alpha ⨻{\;}_{p}\min f.$$

The FLIP scalar multiplication, for p>0, as expressed in Equation (31), is:

(A12) $$\alpha ⨻{\;}_{p}vs.=\frac{1-{\left(\frac{1-(1-p)v}{1-v}\right)}^{\alpha }}{1-p-{\left(\frac{1-(1-p)v}{1-v}\right)}^{\alpha }}.$$

For simplicity, we can compactly denote in the equation above

(A13) $$\pi \left(v\right)={\left(\frac{1-(1-p)v}{1-v}\right)}^{\alpha }.$$

Then, the scalar multiplication can be expressed as:

(A14) $$\alpha ⨻{\;}_{p}vs.=\frac{1-\pi (v)}{1-p-\pi (v)}.$$

The dynamic range can be simply rewritten, in order to obtain Equation (37):

(A15) $$\begin{array}{cc} DR(\alpha ,p,f) & =\alpha ⨻{\;}_{p}\max f-\alpha ⨻{\;}_{p}\min f\\ \; & =\frac{1-\pi (\max f)}{1-p-\pi (\max f)}-\frac{1-\pi (\min f)}{1-p-\pi (\min f)}\\ \; & =\frac{p}{1-p-\pi (\max f)}-\frac{p}{1-p-\pi (\min f)}. \end{array}$$
